# Genomic selection through single-step genomic best linear unbiased prediction improves the accuracy of evaluation in Hanwoo cattle

**DOI:** 10.5713/ajas.18.0936

**Published:** 2019-11-12

**Authors:** Mi Na Park, Mahboob Alam, Sidong Kim, Byoungho Park, Seung Hwan Lee, Sung Soo Lee

**Affiliations:** 1 Animal Breeding and Genetics Division, National Institute of Animal Science, Rural Development Administration, Cheonan 31000, Korea; 2 Poultry Research Institute, National Institute of Animal Science, Rural Development Administration, Pyeongchang 25342, Korea; 3 Division of Animal and Dairy Science, Chungnam National University, Daejeon 34134, Korea; 4 Hanwoo Genetic Improvement Center, NongHyup Agribusiness Group Inc, Seosan 31948, Korea

**Keywords:** Genomic Selection, Single-step Genomic Best Linear Unbiased Prediction (ssGBLUP), Evaluation Accuracy, Proven-bull, Hanwoo Cattle

## Abstract

**Objective:**

Genomic selection (GS) is becoming popular in animals’ genetic development. We, therefore, investigated the single-step genomic best linear unbiased prediction (ssGBLUP) as tool for GS, and compared its efficacy with the traditional pedigree BLUP (pedBLUP) method.

**Methods:**

A total of 9,952 males born between 1997 and 2018 under Hanwoo proven-bull selection program was studied. We analyzed body weight at 12 months and carcass weight (kg), backfat thickness, eye muscle area, and marbling score traits. About 7,387 bulls were genotyped using Illumina 50K BeadChip Arrays. Multiple-trait animal model analyses were performed using BLUPF90 software programs. Breeding value accuracy was calculated using two methods: i) Pearson’s correlation of genomic estimated breeding value (GEBV) with EBV of all animals (r_M1_) and ii) correlation using inverse of coefficient matrix from the mixed-model equations (r_M2_). Then, we compared these accuracies by overall population, info-type (PHEN, phenotyped-only; GEN, genotyped-only; and PH+GEN, phenotyped and genotyped), and bull-types (YBULL, young male calves; CBULL, young candidate bulls; and PBULL, proven bulls).

**Results:**

The r_M1_ estimates in the study were between 0.90 and 0.96 among five traits. The r_M1_ estimates varied slightly by population and info-type, but noticeably by bull-type for traits. Generally average r_M2_ estimates were much smaller than r_M1_ (pedBLUP, 0.40 to 0.44; ssGBLUP, 0.41 to 0.45) at population level. However, r_M2_ from both BLUP models varied noticeably across info-types and bull-types. The ssGBLUP estimates of r_M2_ in PHEN, GEN, and PH+ GEN ranged between 0.51 and 0.63, 0.66 and 0.70, and 0.68 and 0.73, respectively. In YBULL, CBULL, and PBULL, the r_M2_ estimates ranged between 0.54 and 0.57, 0.55 and 0.62, and 0.70 and 0.74, respectively. The pedBLUP based r_M2_ estimates were also relatively lower than ssGBLUP estimates. At the population level, we found an increase in accuracy by 2.0% to 4.5% among traits. Traits in PHEN were least influenced by ssGBLUP (0% to 2.0%), whereas the highest positive changes were in GEN (8.1% to 10.7%). PH+GEN also showed 6.5% to 8.5% increase in accuracy by ssGBLUP. However, the highest improvements were found in bull-types (YBULL, 21% to 35.7%; CBULL, 3.3% to 9.3%; PBULL, 2.8% to 6.1%).

**Conclusion:**

A noticeable improvement by ssGBLUP was observed in this study. Findings of differential responses to ssGBLUP by various bulls could assist in better selection decision making as well. We, therefore, suggest that ssGBLUP could be used for GS in Hanwoo proven-bull evaluation program.

## INTRODUCTION

In 1980s, the Hanwoo performance test (PT) and progeny test (PGT) programs were adopted in the Korean National Evaluation System to assist Hanwoo proven bull selection program. Over time newer breeding tools were utilized for Hanwoo evaluations so that more desirable improvements in phenotypes could be achieved. With more phenotypes being available by 2009, the decade-long older traditional pedigree-based best linear unbiased prediction (pedBLUP) evaluation, *i.e*. single trait animal model, was replaced by the more robust multiple-trait animal model, under the similar pedBLUP architecture. At that time, the main aim of such model adoption was to exploit the existing correlations among traits more efficiently, and thus obtain a better breeding value (BV) profile on animals under such evaluation schemes. Of note, even after the adoption of such advanced and complex models in PT and PGT, there remained other limiting factors with pedBLUP evaluation, such as greater demands for high-quality pedigrees, and the dependence on the number of phenotypes available– especially more records from those of the close relatives. The PGT program, which performs sire performances through the evaluation of its own progenies, is generally known to be an expensive animal improvement scheme due to the higher generation intervals, and its prerequisites for more information from relatives such as progenies, in order to ensure an adequate evaluation accuracy of sires. In PGT, through pedBLUP, an earlier selection of animals was not only difficult but also often required progenies to be sacrificed in the evaluation of most economic traits. Nonetheless, the lack of perfect evaluation accuracy was also an issue with PGT. Yet, animal breeders were limited to choose such expensive breeding tools as possible better alternatives (*e.g*. genotype-based technologies) were not feasible enough to be implemented economically.

Fortunately, over the past decades, the advancement in animal genotyping technologies has made it competitive and more cost-effective. Nowadays, the development in statistical approaches related to genotype-based analyses also made it possible to resolve many of above limitations with pedBLUP. With genotypes, animal BVs can also be estimated easily and more reliably. Note that selection based on animal’s genomic information, also known as genomic selection (GS [1], has already been implemented in many countries. The higher accuracies with GS were reported by many recent studies [2,3]. With genotypic information on progeny and parents, and through genomic BLUP (GBLUP), the earlier selection of animals is made possible, and therefore can greatly reduce the generation interval and cost of production.

To illustrate briefly, it is worth noting that GS essentially allows the prediction of animal’s genomic estimated breeding value (GEBV) through a genomic relationship matrix (GRM), which is based on the genotype information of the animals and using a GBLUP method. For GBLUPs, two widely-used methods are also available, *e.g*. a multi-step and a single-step method [4]. The single-step GBLUP (ssGBLUP) method is more sophisticated, yet easier to implement than the multi-step method. The ssGBLUP also allows the estimation of BVs for all animals at the same time.

We observed that there has been a great momentum for the large scale implementation of GBLUP and GS in various livestock species in the recent years [5,6]. This is thought to be due to the relative advantages of GBLUP regarding more accurate estimation of an animal’s genetic merit as compared to the parent-average based traditional method. Note that most applications of GS as observed are in dairy cattle, whereas in beef cattle this is still an emerging technology [7]. However, the National Genetic Evaluation (NGE) system of Hanwoo cattle has recently integrated a multi-step GBLUP based GS approach in a limited scale, alongside the pedBLUP, with a consideration that it would optimize breeding decisions and selection of young candidate bulls with much greater accuracy than before. However, the impact of a multi-step GBLUP based GS approach could be limited due to its technical limitations, together with the shortcomings of Hanwoo population. A practical implementation of ssGBLUP could be an appropriate choice to solve some of those challenges. Until now, most of the ssGBLUP reports and its comparison to pedBLUP was conducted in relatively smaller samples of Hanwoo cattle. In this study, we investigated the impact of ssGBLUP using a much larger Hanwoo population to obtain a more robust comparison of above methods. In this study, we also compared the improvements of evaluation accuracy in specific bull-types of Hanwoo so that it could assist in selection decision making process.

## MATERIALS AND METHODS

### Animal phenotype

In this study, yearling weight and carcass trait measures were recorded on the males of Hanwoo cattle that were raised under Korean National Improvement System. A total of 9,952 bulls, born between 1997 to 2018 under proven-bull selection program, were recorded for phenotypes. Phenotyped bulls were considered to be in one of three categories such as those of young male calves (YBULL, ~6 mo of age), young candidate bulls (CBULL, selected from a pool of YBULL) and, progeny tested bull (PBULL). All CBULL bulls were recorded for yearling weight at 12 months of age (WT12) for PT program. Carcass traits studied in this study were carcass weight (CWT), backfat thickness (BFT), eye muscle area (EMA) and marbling score (MS). Details on recorded animals are presented in Table 1. Animal procurements for PT and PGT programs were described in detail by an earlier study [8]. Note that the animals that we studied here were between 25 and 74 (PT) and, between 36 and 63 (PGT) batches, where batch numbers were indicative of the year and season of birth of bulls [8].

### Genotypic data, quality control, and genotype imputation

Genotypic data on 7,387 Hanwoo males were generated in two batches using a high-density Illumina BovineSNP50K BeadChip (Illumina, Inc., San Diego, CA, USA) array as per the standard protocol. Then, two genotype datasets were combined into one based on common autosomal SNPs (n = 52,825), followed by a population-based genotype imputation step using FImpute 2.0 software package [9]. That imputed dataset was further constrained for several commonly applied genotype quality control options through PREGSF90 software package [10]. The criteria for SNP exclusions were animals with parentage errors (genotype-based), presence of monomorphic allele, a less than 5% minor allele frequency, and a less than 90% genotype call-rate. An animal was also completely removed from the genotype dataset if its genotype missing rate exceeded 10%; after which 39,308 SNP markers and 7,374 animals were available for further analysis.

### Animal pedigree

A pedigree on studied animals was collected from Korea Animal Improvement Association (KAIA). The pedigree, related to animals with phenotypes and genotypes, included 67,802 animals and extended up to the maximum of 14 ancestral generations. A total of 19,260 animals were found as inbred in the dataset. This pedigree also included 1,393 sires, 46,202 dams, and 516 full-sib family groups (with an average family size of 2.1). Note that about 95% of the inbred animals showed lower inbreeding rates (0% to 5%). Although the highest inbreeding coefficient in the study was 0.31, the average coefficients in the whole population and within the inbred animals were 0.004 and 0.015, respectively. We also calculated the average relatedness of all animals using the pedigree data. Mainly, two software packages, *i.e*. PEDIG [11] and CFC 1.0 [12] were used in this step; of which the former was used for preparing the pedigree data, and the later was used in determining pedigree structure, inbreeding coefficients and average pedigree-relatedness of the animals.

### Estimation of EBV by pedBLUP

A conventional BLUP method [13] based multiple trait animal model analysis was performed to obtain EBV estimates of traits; which was also the model practiced in NGE system until very recently. The dataset for animal model fit included 36,225 records of all five traits. Animal’s batch number (B), birth location (L), test station (T), and slaughter date (S) were combined into two composite fixed effects, *i.e*. BLT and BTS. The fixed effect of BLT was fitted with WT12, whereas BTS with carcass traits only. The fixed covariate of age at slaughter was also fitted with carcass traits. As for fitting random effects, the additive genetic effect of the animal was the only random genetic component in the model. We, then, estimated animal BVs and standard errors of prediction (SEP) using BLUPf90 software package [14]. The mixed model equation used for pedBLUP using matrix notations was **y** = **Xb** + **Zu** + **e**, where **y** is the vector of traits, **b** is the vector of fixed effects and covariates, **u** is the vector of random effects (additive genetic), **e** is the vector of random residual effect, and **X** and **Z** are the respective design matrices relating observations to the fixed and random effects **b** and **u**. The variance structure of **u** and **e** were assumed as var (**u**) = **G_0_** ⊗ ***A***, var (**e**) = **R_0_** ⊗ ***I***, where **G_0_**, **A**, **R_0_**, and **I** were the additive genetic (co)variance matrix between traits, the pedigree relationship matrix, the residual (co)variance matrix between traits and the identity matrix, respectively.

### Estimation of genomic estimated breeding value by ssGBLUP

The estimation of GEBV was performed by ssGBLUP method through fitting factors as described for pedBLUP analysis. However, instead of an A^−1^, the ssGBLUP used an H^−1^ [4] matrix, which is an inverse matrix derived from the relationship matrices based on pedigree and genotype datasets. Thus, the mixed model equation for ssGBLUP was

$$\begin{array}{l} \left[\begin{array}{cc} X^{\prime }R^{-1}Z & X^{\prime }R^{-1}Z\\ Z^{\prime }R^{-1}X & Z^{\prime }R^{-1}Z+H^{-1}\frac{1}{\sigma_{u}^{2}} \end{array}\right]\;\left[\begin{array}{c} b\\ u \end{array}\right]=\left[\begin{array}{c} X^{\prime }R^{-1}y\\ Z^{\prime }R^{-1}y \end{array}\right],\\ H^{-1}=A^{-1}+\left[\begin{array}{cc} 0 & 0\\ 0 & \tau G^{-1}-\omega A_{22}^{-1} \end{array}\right] \end{array}$$

where **G** is a genomic relationship matrix, τ and ω are adjustment for **G** and **A** respectively. Following VanRaden [1], G was calculated as:

$$G=\frac{{\mathrm{WDW}}^{\prime }}{2\;\Sigma_{i=1}^{n}p_{i}(1-p_{i})},$$

Where *p_i_* is the allele frequency at locus *i* in all genotyped animals, $2\;\Sigma_{i=1}^{n}\;p_{i}(1-p_{i})$ is a normalizing constant [15] that sums expected variances across markers scaling **G** towards the **A** matrix [1], **D** is weight for each locus (I if same variance assumed), **W** is a design matrix as follows:

$${\text{w}}_{\text{ii}}=\{\begin{array}{ll} 0-2p_{j}, & homozygous\\ 1-2p_{j}, & heterozygous\\ 2-2p_{j}, & homozygous \end{array}$$

Both **G** and **H** matrices were derived using software default parameter settings for τ (0.05) and ω (0.05) by PREGSF90 at runtime. Finally, animal BV solutions and their SEPs were estimated by BLUPF90 using appropriate options for the software runs.

### Prediction accuracy of breeding values

Theoretically, the accuracy of (G)EBVs are referred to as the correlation between estimated (genomic) breeding values of animals and their respective true breeding values (TBV), where TBV is generally unknown for any given population [3]. As a result, several methods for approximation of accuracy were proposed by many authors to overcome the limitation of TBV– even though in practice none were found to be completely unbiased [3]. For simplicity reasons and the ease of comparison across studies for Hanwoo and other breeds, we reported the efficacy of ssGBLUP model GEBVs through two commonly used methods.

The first estimate of the accuracy of GEBVs is also referred to as the prediction accuracy of the model, where GEBVs derived through ssGBLUP are directly compared with EBVs from pedBLUP for all animals. This method of estimation was based on the report from Daetwyler et al [16], in which the use of more readily available animal EBVs was proposed instead of using the theoretically existent TBVs. In this method, the estimation of prediction accuracy was rather expressed as *r*_M1_ = *Cor_EBV,GEBV_*, where r_M1_ was a Pearson’s product-moment correlation coefficient between the GEBV and EBV, which we could easily obtain through ssGBLUP and pedBLUP models in this study, respectively. A possible advantage of this approach would be the ability to compare the current outcomes with other studies that were already available. Another benefit of this estimate was the ability of an overall assessment for the expected accuracy of animals, where all available samples was considered at a time, and without further differentiation for number of information available. In other words, the accuracy of estimation could be interpreted on a population level.

Contrarily, the second type of accuracy was calculated at each individual level. In this method, the prediction error variance (PEV) of both EBV and GEBV were estimated for each animal and the respective trait. Such PEVs were then used for the calculation of accuracy for each EBV and GEBV estimates per animal, respectively, using the equation, $r_{\text{M}2}=\sqrt{\left(1-\frac{PEV}{V_{A}}\right)}$. The r_M2_ and PEV in the equation denoted the accuracy and error-variance estimates for each animal, respectively. Note that each PEV estimate can be directly obtained for all individuals from the inverse of the coefficient matrix of the mixed model equation i.e. the MME matrix, as shown earlier by [1,13]. For PEV, we first obtained the standard error of prediction (or the square root of PEV) for each animal and trait through setting proper options while BLUPF90 program runs, which was finally converted to a PEV estimate. The only additional component in the equation, *i.e*. V_A_ or additive genetic variance of each trait, was estimated separately through REML method for the same dataset but excluding genotype information by REMLF90 [14] software package. All r_M2_ measures for respective traits were compared to animals based on the type of bulls, level of pedigree relatedness, and type of information available on animals, *e.g*. phenotyped or genotyped animals etc.

### Estimation of genetic parameters

Genetic parameters and correlation on traits were also reported in the study. For these estimates, the estimation of variance and covariance components were carried out using the same dataset and an animal model fitting of the same set of fixed and random effects as described above under the model description for pedBLUP evaluation. The approximation of (co)variance components were obtained through a REML approach using REML90 software. Total phenotypic variance was calculated as $\sigma_{p}^{2}=\sigma_{a}^{2}+\sigma_{e}^{2}$, and the heritability estimate was derived as $h^{2}=\sigma_{a}^{2}/\sigma_{p}^{2}$, where $\sigma_{a}^{2}$ and $\sigma_{e}^{2}$ were the estimates of additive genetic variance and random residual variance, respectively. Also, the correlation between traits were calculated from the derived (co)variance estimates from the animal model analysis.

## RESULTS

### Descriptive statistics on phenotypes

Descriptive statistics on five phenotypic traits according to info-type such as PH+GEN (both phenotyped and genotyped), and PHEN (phenotyped only) are presented in Table 2 and 3, respectively. The observed average yearling weight or WT12 within PHEN category was slightly higher than those of the PH+GEN category of animals. The PHEN group also showed more spread (*i.e*. standard deviation) alongside the higher variation in their phenotypic data. The same animal subset conversely showed relatively higher averages and lower variations for all carcass traits than those of PH+GEN, especially in their BF and MS traits. It is found that phenotypic measures in the present study were either in general agreement with a few earlier reports on Hanwoo cattle [17,18] or differed slightly being higher than others [19,20]. We also observed some noticeable differences in sample sizes among studies and that might explain some of the estimation variations with our study and other reports. We used a larger sample size as compared to other reports on Hanwoo cattle. The time of reporting is also worth mentioning in the sense that there had been tremendous efforts regarding planned breeding and selection appraisals in Hanwoo cattle to enhance performances over the past few decades, and thus, resulting in positive gains in many economic traits. Therefore, some higher phenotypic measures in important economic traits as in this study might not be quite unexpected either.

### Genetic parameter estimates

Heritability (h^2^) and correlation estimates for five traits are given in Table 4. The h^2^ estimates of WT12 and CWT were 0.26 and 0.35, respectively. Moderate to higher h^2^ estimates were observed in EMA (0.44), BFT (0.46), and MS (0.56) traits as well. Genetic (r_g_) and phenotypic correlations (r_p_) of WT12 were the highest with CWT (r_g_: 0.70 and r_p_: 0.71). CWT was moderately correlated with EMA (r_g_: 0.55). The r_g_ between EMA and BFT was negative, *i.e*. −0.24. The MS-r_g_ was low and positive with CWT and EMA, but low and negative with WT12 and BFT. However, all r_p_ estimates were positive except for the r_g_ between BFT and MS traits.

### Accuracy of evaluation by animal population

The accuracy estimates of single-step genomic evaluations using two earlier described approaches (r_M1_ and r_M2_), using the population dataset, are shown in Table 5. The r_M1_ estimates (the correlation between individual’s GEBV and EBV) were generally higher among traits and showed less variability, *i.e*. 0.90 to 0.96. This r_M1_ of MS was the lowest among all five traits, whereas the highest for WT12 and EMA. The r_M2_ estimates (derived from the coefficient matrix of MME), in contrast, had a higher variability range (0 to 0.95), which also showed noticeably lower averages (0.40 to 0.45) in all traits. Those averages among five traits and between two evaluation methods (ssGBLUP and pedBLUP) were generally not the same but close and demonstrated a slight improvement of 2% to 4.5% by ssGBLUP over pedBLUP. For traits under concern, MS had the lowest average r_M2_ estimates, which was also the highest improvement by ssGBLUP according to whole population. Both BFT and EMA realized over 3% increment in BV accuracy by ssGBLUP, and WT12 had the lowest improvement by 2%. The overall performance of ssGBLUP at the population level, therefore, to be considered as some improvement over the pedBLUP evaluation.

### Accuracy of evaluation by animal’s info-type

**Figure 1:**
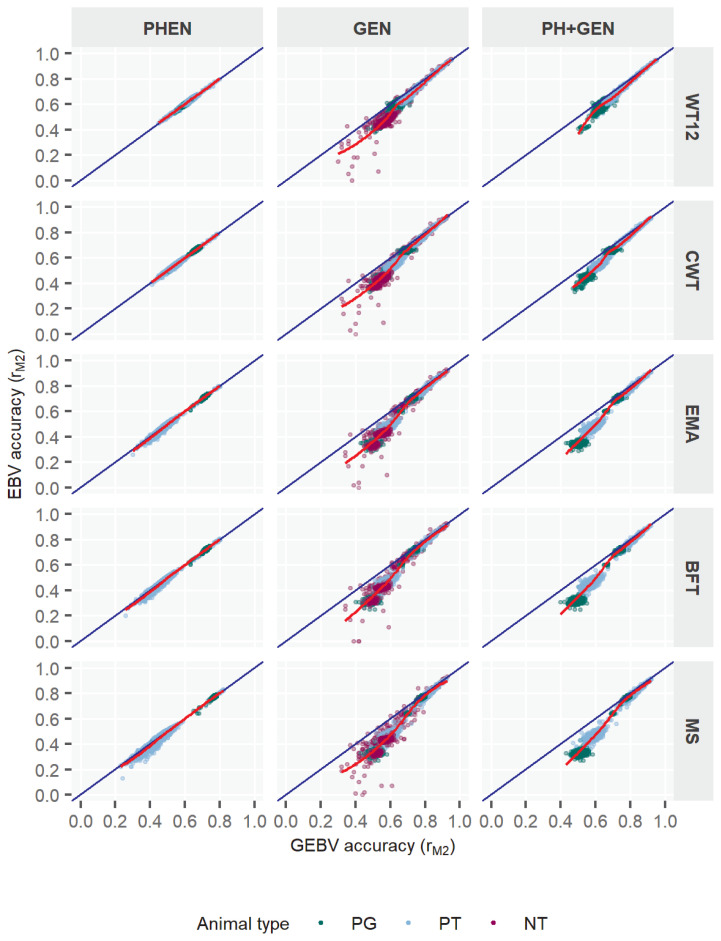
Accuracy of genomic estimated breeding value (GEBV) for five Hanwoo traits by different types of data subsets. EBV, estimated breeding value; PHEN, phenotyped-only; GEN, genotyped-only; PH+GEN, phenotyped and genotyped; WT12, weight at 12 months; CWT, carcass weight; EMA, eye-muscle area; BFT, backfat thickness; MS, marbling score; PG, bulls from progeny test; PT, bulls from performance test; NT, bulls not tested.

The accuracy by animal info-type categories, *i.e*. PHEN, GEN and PH+GEN, are presented in Table 6 and Figure 1. In this study, r_M1_ estimates based on three info-types ranged between 0.89–0.99 among five traits, where PHEN showed the higher estimates (0.96 to 0.99), followed by PH+GEN (0.91 to 0.93) and GEN (0.89 to 0.92) group estimates. Although these estimates (r_M1_) were somewhat similar among three info-types, there was some noticeable differences in r_M2_ estimates among these animal categories, irrespective of ssGBLUP or pedBLUP estimates. The average r_M2_ within info-types were also significantly lower than the average r_M1_ estimates across all methods of evaluation. Unlike r_M1_ estimates, the r_M2_ estimates indicated relatively higher accuracy in PH+GEN bulls, bulls that provided both genotypic and phenotypic information to the models of interest. It is also clear that the accuracy ranges in PH+GEN bulls were narrower, and all estimates were relatively higher (Figure 1). To illustrate the fact, MS-r_M2_ estimates in PH+GEN group were found as the narrowest (0.43 to 0.92), which also indicated about relatively larger lower and upper bounds of estimate as compared to those of either PHEN (0.23 to 0.82) or GEN (0.32 to 0.93) estimates; which also concurred with other traits. Both evaluation models showed similar patterns in this manner. As far as the improvement by ssGBLUP was concerned, the r_M2_ estimates indicated some none-to-little positive changes (0% to 2%) among the BV estimates of all studied traits. This indicated that PHEN bulls, even if they were included and evaluated under ssGBLUP, were unable to capture much of the benefits from the single-step method. This further emphasized the importance of type of information in the dataset. In this regard, we found substantial improvements in accuracy within GEN and PH+GEN bulls. Both these later bull categories (with genotype information) obtained GEBVs which were seemingly more reliable by 8.1%–10.7% and 6.5%–8.5%, respectively, to their pedigree based EBVs. Regarding trait BV responses to ssGBLUP evaluation, the GEBV estimates of BFT, MS, and EMA showed more reliability improvements than was obtained by WT12 (Table 6). It is also to note that the difference in r_M2_ between evaluation methods was comparatively higher with GEN than other two bull groups. This might be because GEN group evaluations become more accurate when genotype information is used instead of evaluating no phenotypic information at least from those bulls, which are also mostly young candidates in the population. Thus, based on bull’s info-type, it could be stated that while animals with both phenotype and genotype information obtain the highest accuracies, whereas phenotype-only animals gain the lowest among all, the most significant outcomes are to be attributed to those bulls that are yet young (*i.e*. no phenotypes) but have genotypes. Additionally, Figure 1 also shows how bulls, included under different programs (*i.e*. mainly in PT and PGT) and under info-type categories in the study, could vary distinctly by distribution of estimates among them. PGT bull accuracies in GEN and PH+GEN mostly appeared in small clusters both at the lower and higher end of estimates. However, PT bulls showed less clusters and estimates were spread at wider ranges. The relatively lesser spread of estimates outside the 1:1 regression line fewer in PHEN category for PT and PGT also reflected their similarities across traits. Another important finding (Figure 1) was related to those of PGT bulls with low accuracies. This could indicate that those bulls, even though progeny-tested, could perform poorly and affect the improvement if selection is based on them.

### Accuracy of evaluation by bull-type

**Figure 2:**
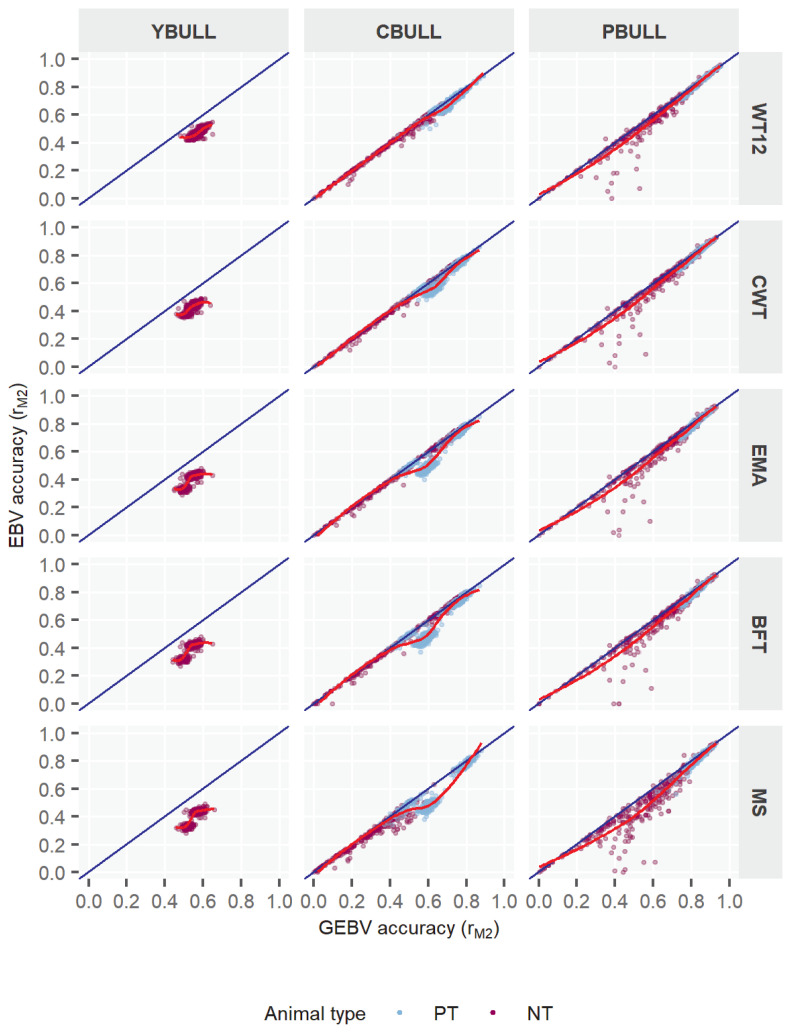
Accuracy of genomic estimated breeding value (GEBV) for five Hanwoo traits by different type of bulls. EBV, estimated breeding value; YBULL, young male calf; CBULL, young candidate bull; PBULL, proven bull; WT12, weight at 12 mo; CWT, carcass weight; EMA, eye-muscle area; BFT, backfat thickness; MS, marbling score; PT, bulls for performance test; NT, bulls not tested.

Table 7 illustrates the accuracy by three bull types (YBULL, CBULL, and PBULL) as categorized in this study. For all traits, the r_M1_ estimates of evaluation ranged between 0.70–0.96 among bull-types, where CBULL and PBULL estimates were much higher than that of YBULL. The r_M1_ estimates in YBULL were between 0.70 and 0.75, and in other two categories those were between 0.89 and 0.96. Comparing r_M1_ to r_M2_, the former was much larger than the later, like the pattern observed earlier among info-types. The only distinction, however, was that unlike the narrower range obtained within info-types, r_M1_ range in three bull-types was much broader, *i.e*. 0.70 to 0.96. Regarding BV estimation methods, the r_M2_ estimates also differed among bulls-types. As observed, PBULL range of mean accuracy (r_M2_) was 0.70 to 0.74 and the highest in the study. GEBV accuracy of other two bull-types were somewhat similar though which were 0.55 to 0.62 in YBULL, and 0.54 to 0.57 in CBULL. The pedBLUP-based mean accuracies were also lower than those based on ssGBLUP in all traits. Due to the very similar patterns in ssGBLUP and pedBLUP accuracies across bull-types, it was obvious that the improvements by ssGBLUP over pedBLUP would differ across bull-types as well. We, therefore, observed substantial differences in improvements across bull-types. Interestingly, YBULL which gained a lowest accuracy in ssGBLUP for all traits also showed the most positive improvements in accuracy by +21.3% to +35.7%. Accuracy improvement in CBULL remained moderate between +3.3% and +12.2% across traits. PBULL, in contrast, that obtained the highest r_M2_ by ssGBLUP also showed the lowest positive changes in accuracy (2.8% to 6.1%) with respect to pedBLUP. It was also clearly observed that improvements in accuracy by traits was different among bull-types. Among traits, MS showed most positive improvements by 6.1% to 35.7% by ssGBLUP, followed by 4.3% to 34.1% in EMA and 4.3% to 31.7% in BFT. Improvements in CWT and WT12 by ssGBLUP were also noticeably higher (Table 7). More insights into bulls that were included in the programs also showed similar patterns as for info-types. However, the clusters of YBULL estimates might indicate that benefits of ssGBLUP was also limited to those animals (also mostly NT or non-tested type). Because their evaluations were only based on the genotypes for which variations in the population were supposed to be smaller as Hanwoo is a closed breed. With CBULL or PBULL groups, such estimates were widespread and interesting as well, especially in the PBULL, because those bulls added more information through phenotypes and genotypes. The advantages of ssGBLUP became clearer through their distribution on graphs for most traits, where plotted low to medium range values were mostly offset from the 1:1 regression line. This further emphasized the expected benefits of ssGBLUP for those animals in terms of higher accuracy. Interestingly, even though we have observed relatively smaller overall improvements in PBULL by ssGBLUP (Table 7) earlier, particularly the NT bulls among them did show significant improvement (Figure 2) under the same evaluation for all traits. Note that NT bulls in practice may or may not directly contribute to the improvement programs as they could be either young or older parents, but their inclusion in ssGBLUP model with an improved accuracy might lead to better overall evaluations of others.

**Figure 3:**
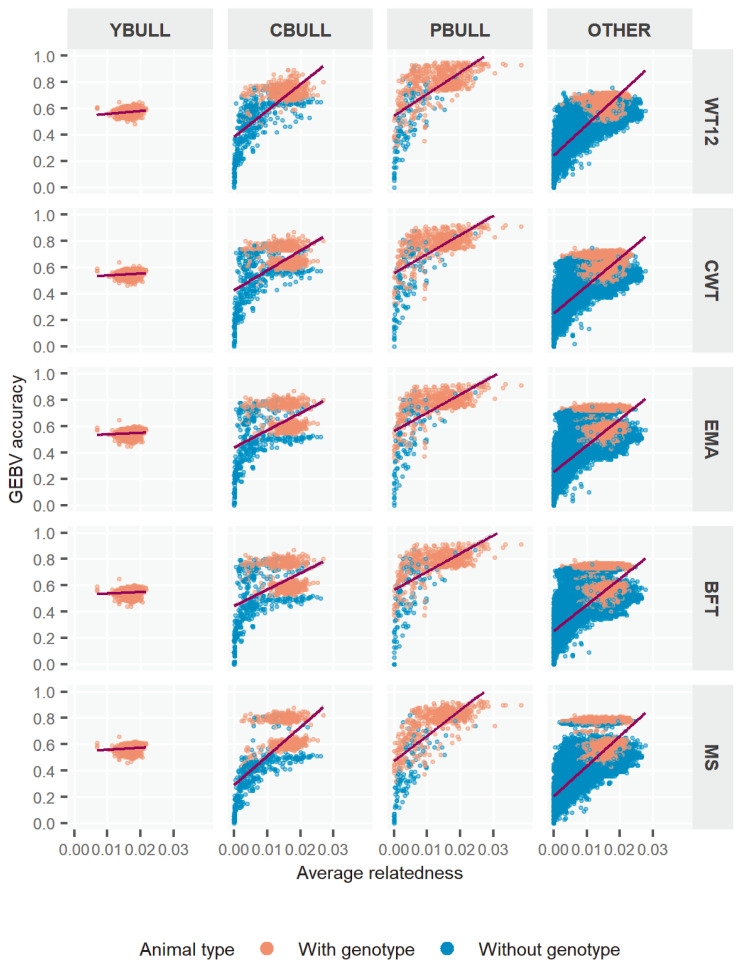
Accuracy of genomic estimated breeding value (GEBV) by pedigree relatedness in different types of bulls for five Hanwoo traits. YBULL, young bull calf; CBULL, young candidate bull; PBULL, proven bull; OTHER, other bull; WT12, weight at 12 months; CWT, carcass weight; EMA, eye-muscle area; BFT, backfat thickness, MS, marbling score.

Figure 3 further plots the GEBV accuracy according to the existing average relationships among bull-types. We calculated the relationship for each animal using the respective coefficients related to individual from the NRM. In YBULL, the relatedness and r_M2_ was not correlated and rather unexciting. With CBULL, PBULL and the rest, we observed very low accuracy when relatedness was none or little, *i.e*. 0 to 0.01, whereas it obtained medium to high r_M2_ at relatedness of 0.02 and onwards, overall. Further subdivision of each bull-type into genotyped and non-genotyped animals showed some interesting outcomes. Most lower estimates of r_M2_ were mostly associated with non-genotyped bulls rather than genotyped ones. Especially, genotyped animals had medium to high and mostly consistent r_M2_ estimates across relatedness levels. Although, this tends to be little spread with PBULL, but others followed somewhat similar trends. Estimates across traits by relatedness also tend to be somewhat robust among bull-types. These r_M2_ outcomes clearly explained that genotyped bulls, irrespective of bull-types, were better evaluated by ssGBLUP, which could be simply because genotype information maximized the animal contributions through GRM more accurately where pedigree only was unable to capture. Also note that ssGBLUP is designed to weight genotyped animal contributions more appropriately through model parameters. In ssGBLUP model, non-genotyped animals that mainly relied upon pedigree relationships to capture variations in phenotypes eventually assessed GEBVs with lesser accuracies– hence the wider range of estimates.

## DISCUSSION

### Genetic parameters

For genetic parameters estimates, this study coincided with Shin et al [18], who reported very similar results in Hanwoo cattle, *i.e*. CW (0.36), EMA (0.44), BF (0.48), and MS9 (0.58) using pedigree BLUP dataset. Little disagreements were observed from Choi et al [21] with their slightly lower pooled h^2^ in yearling weight (0.25), carcass weight (0.29), longissimus muscle area (0.38), and backfat thickness (0.45), but a slightly higher in MS (0.62), which could be due to their relatively larger datasets. We also found subtle differences with Park et al [20] with their slightly higher h^2^ for yearling weight (0.30), backfat (0.50) and MS9 (0.63) or with slightly lower h^2^ for carcass weight (0.30), but with a similar EMA (0.42). The h^2^ of CWT in Hanwoo from earlier reports [21–26] was also somewhat consistent with our results. Moderate BFT h^2^ in this study was deemed consistent with Hwang et al [23] Lee and Kim [27]. Such subtle to greater disagreements were likely as sample sizes and model differences were somewhat obvious among studies. For example, Choi et al [28] showed h^2^ for IMF as 0.55 and 0.69, using GRM and NRM, respectively. They reported that residual variances increased by GRM based method, whereas BV variance increased by NRM based method. That could be equally applicable for correlation estimates. Overall, there were no great differences among estimates across studies.

### Differences in BV accuracy by evaluation models, info-type, bull-type, and relatedness

In this study, we analyzed five important beef traits in Hanwoo cattle and compared the evaluation accuracy of these traits with respect to pedBLUP and ssGBLUP. Our aim in this study was to analyze the impact of both pedigree and genomic information driven prediction methods on the BV estimation among several bull categories, which were raised under the proven bull selection program. As it has been stated earlier, since the implementation of GS in Korea for Hanwoo proven bull selection is very recent, the practical impact on different animal groups is yet to be understood properly. Also note that GS has been widely applied in dairy cattle and proved to be successful. For beef cattle evaluation, however, this has not been tested extensively so far, and there are very limited resources to compare. This indicates that it would be equally challenging to verify outcomes in the light of beef cattle, given that GS under implementation can also differ significantly among breeds and as per breeding objectives.

With various model accuracies, in general, the present study revealed a noticeable increase in the BV accuracy by ssGBLUP when compared to pedBLUP evaluation. Different animal groups as well as traits also reported differently in this manner. Our report observed the improvement by genomic model to be as high as 35% for MS in YBULL. Some earlier reports on Hanwoo cattle also concurred with the present study. A recent ssGBLUP study in Hanwoo cattle by Shin et al [18] presented greater agreements in various traits estimates which utilized a smaller reference population of 348 cows and 3,820 steers. According to their report, the accuracy increased by ssGBLUP over pedigree-BLUP for specific traits such as CWT, BFT, EMA, and MS were 22.9%, 12.28%, 11.14%, and 8.69%, respectively. Although our estimates were not in complete agreement with respective traits [18], it did coincided greatly in that genomic evaluation by ssGBLUP as an effective method to enhance BV accuracies noticeably over the traditional ones. Further support with our study was asserted by Choi et al [28] who reported an improvement by at least 1.5 times more GEBV accuracy for intramuscular-fat predictions by multi-step GBLUP methods (using different GRMs) with respect to the traditional approach. Another report on GBLUP for EMA, BFT, and MS traits in the same breed by Lee et al [29] also stated accuracy increments by +0.16 to +0.19 points, whereas Lee et al [19] in another study provided evidence of the positive impact due to addition of SNP data to the model, and appeared to be in general agreements with our results. Our report also showed a non-linear relationship between GEBV and EBV (Figures 1, 2), with which Badke et al [30] also concurred. The lower correlations between pedigree and genomic models (0.28 to 0.45) shown by Choi et al [28] was also an agreement with the present report. Being two models less correlated to each other also means that accuracies for an individual using ssGBLUP is less likely to be similar with pedBLUP and less likely to be plotted on the 1:1 regression line as shown in Figure 1 and 2. Overall, the present study and the collected evidences from earlier Hanwoo reports basically supported that any advanced methods using genotypic data such as single-step methods or any other techniques related to GS are generally superior to traditional methods, and therefore, it can benefit in the Hanwoo cattle improvement substantially.

In this study, we also reported the differences in the animal evaluations based on certain classification such as info-type or bull-type. Evidently, some distinct responses from animals’ accuracies due to info-types were clearly noticeable with ssGBLUP and not so much with pedBLUP. Not only that, the average accuracy for most info-types were uplifted by ssGBLUP, the baseline estimates also improved, and therefore, it decreased the spread between the lowest and highest estimates. For instance, we showed the lowest estimate in PH+GEN with ssGBLUP was higher by +0.15 points than pedBLUP, so as by +0.34 in GEN. Further investigation of the results (Table 6, Figure 1) revealed that bulls that were poorly predicted by pedBLUP, *i.e*. low to medium r_M2_, had also been mostly benefited by ssGBLUP. Our results on bull-type comparisons were also equally appealing and displayed similar patterns as with info-types. We found that the most benefited bull-type was YBULL with an accuracy improvement between 21.3% to 35.7% by ssGBLUP among the five traits. The responses in CBULL and PBULL were somewhat lower (2.8% to 12.2%) among traits but not negligible. Especially the lower improvement rates for PBULL was neither unexpected, as even with a pedBLUP proven bulls would capture most of the heritable genetic variances due to their large progeny pools in the dataset. Therefore, any improvement by ssGBLUP to PBULL, even if it is small, would be considered as significant. When both info-type and bull-types were investigated together, it was not surprising to find that most YBULL and CBULL were also GEN or PH+GEN bulls. With this it became more obvious that the higher GEBV accuracies in such genotyped young or candidate bulls were desired, as the inclusion of genotype information to the model (through GRM) could predict animal BVs with increased accuracy [1,28,30]. Results for PH+GEN on five traits were promising too. However, for PHEN bulls, we consistently obtained lower estimates and that could be related to the fewer PHEN samples in the data. Because an access to large quantities of phenotypic information is important to attain desirable gains in animal evaluation accuracy [31].

With animal relatedness, we noticed some relationships between relatedness and r_M2_ estimates. This might indicate that those of closely related animals were benefited by ssGBLUP. In this regard, some earlier reports agreeably showed evidences for the positive association between closely related animals and their higher GEBV accuracies [30,32]. Nonetheless, there were multiple reports as well which claimed that even unrelated [32] or distant relatives [30] could obtain higher evaluation accuracies. As a precondition, however, Badke et al [30] emphasized a sufficient genetic diversity in the reference population for such accuracy increases in unrelated animals. From all these reports, it could be summarized that rather than using animal relatedness to increase accuracy, it would be worth considering the data quality instead that would provide greater genetic variability and desired selection responses through GS.

### Importance of ssGBLUP based GS in Hanwoo evaluation

We already mentioned that adoption of GS is very recent in Hanwoo cattle development. Currently, GS is performed at a limited scale in the Hanwoo Proven Bull Selection Program. To better illustrate, we can summarize the proven-bull selection program into four subsequent phases such as i) production of young bull-calves by design mating of PBULL and adult cows from various designated farms; ii) selection and procurement of some of those young bull-calves as young candidate bulls and performance testing; iii) production of progeny from (by design mating) using performance-tested young bulls and adult cows from designated farms, and iv) selection of those performance-tested young bulls as proven bulls via progeny testing. GS is performed between phase 1 and 2, when young (candidate) bulls are to be procured from a pool of young calves (generally under the age of 6 mo). Previously, a pedigree index, assisted by an appearance inspection process, was the main tool for young (candidate) bulls’ selection. Now, through GS, as soon as the genotype of all those young males are known, their GEBVs are calculated through summation of total SNP effects. Note that these SNP markers effects are rather based on their parents NGE which were performed earlier through a multi-step GBLUP method. Also, the direct genetic value (DGV) or GEBV is used instead of an EBV to calculate performance index of animals in various phases of bull selection. However, the selection of the young males is a very important step as they will become the proven bulls for the next generations. Therefore, the selection of the candidate (bull) pool is now more reliable and accurate than before due to GS in young males. It is also possible to avoid all additional testing steps (e.g., appearance or performance) and select animals much earlier in their lifetime; thereby, reducing generation intervals as well as the cost of animal productions.

Although the current GS is an upgrade to the earlier methods, the real benefits from GS deemed rather limited. Firstly, the recent practice of GS in Hanwoo is limited by scale. To reap the full benefit from GS, it could be used in Hanwoo preselection processes, such as PT and PGT phases. This might increase the genetic gains in major selection traits at a much-desired extent, especially in MS. Secondly, the multi-step GBLUP based GS approach itself poses some technical limitation. This is because the Hanwoo reference population that is used in this purpose is very small (if compared with other cattle breeds). For a multi-step GBLUP, a larger reference population for bull evaluation is essential [33]. Nonetheless, this method is only limited to the evaluation of animals having both genotypes and phenotypes. As a result, many animals that have only phenotypes cannot contribute in the estimation of SNP effect and, thereby, in DGV estimation of Hanwoo cattle. A more realistic approach would be to use a method, such as ssGBLUP, which allows all the animals in the population to participate in the evaluation process. Subsequently, additional bulls in the evaluation would help ensuring much higher accuracies. Furthermore, the evaluation through ssGBLUP on genotyped-only animals has other additional advantages. With a multi-step or 2-step GBLUP model, any genotyped young bull can no longer influence the evaluation of other animals later if it is culled after evaluation. Through ssGBLUP, these culled (genotyped) bulls can participate in the subsequent evaluations, and further influence the evaluation of other animals. So, our believe is that an adoption of a much simpler ssGBLUP-based GS would be more practical, especially when the multi-step methods efficiencies could be limited due to various preconditions that deemed difficult to fulfill in Hanwoo population.

## CONCLUSION

The ssGBLUP is generally appreciated because of its unified framework for more accurate genomic evaluations of animals. To date, its application in many species also produced better results. Our results were largely in agreement with these previous studies as well, where ssGBLUP performed noticeably better than pedBLUP. Increase in accuracy through ssGBLUP was also substantial in most of bull sub-groups, especially to those without phenotypes. As young bulls are the next generation of parents, future selection decisions based on ssGBLUP is deemed promising as baseline accuracies were significantly improved in this study. Differences in traits improvement regarding ssGBLUP were also indicated in our outcomes. We also provided evidence how the currently implemented multi-step GBLUP based GS in Hanwoo might face challenges. We believe that this study provided strong evidence for the success of ssGBLUP in Hanwoo national evaluation as a tool for GS.

## Figures and Tables

**Table 1:** Details on animal subsets and distribution of bulls in the data

Subset	Phenotype1)	YBULL2)	CBULL2)	PBULL2)	Total (N)
PH+GEN (Genotyped and phenotyped)	WT12 only	0	504	348	2,211
	WT12 and CT	0	0	0	3,656
PHEN (Phenotyped-only)	WT12 only	0	108	1	8,331
	CT only	0	0	0	1,837
	WT12 and CT	0	0	0	1,381
GEN (Genotyped-only)	-	758	34	208	1,417
Others	-	0	255	106	48,879

1) WT12, weight at 12 mo, CT, carcass traits (carcass weight, eye-muscle area, backfat thickness, and marbling score).

2) YBULL, young bull-calves (~6 mo of age); CBULL, young candidate bulls or calves (6 to 12 mo of age); PBULL, proven bulls (~48+mo).

**Table 2:** Descriptive statistics on phenotypes in genotyped and phenotyped (PH+GEN) data subset

Trait	N	Mean	SD	Min	Max	CV (%)
WT12 (kg)	5,867	350.53	54.12	144	561.5	15.4
CWT (kg)	3,656	371.63	42.07	160	562	11.3
EMA (cm^2^)	3,656	81.65	8.82	41	130	10.8
BFT (mm)	3,656	9.71	3.82	1	35	39.3
MS (1–9)	3,656	3.58	1.62	1	9	45.3

SD, standard deviation; Min, minimum value; Max, maximum value; CV, coefficient of variation; WT12, weight at 12 mo; CWT, carcass weight; EMA, eye-muscle area; BFT, backfat thickness; MS, marbling score.

**Table 3:** Descriptive statistics on phenotypes in phenotyped-only (PHEN) data subset

Trait	N	Mean	SD	Min	Max	CV (%)
WT12 (kg)	9,712	355.94	41.22	176	565.5	11.6
CWT (kg)	1,381	355.99	39.25	158	488	11.0
EMA (cm^2^)	1,379	79.62	8.74	42	121	11.0
BFT (mm)	1,381	9.57	3.97	2	28	41.5
MS (1–9)	1,381	3.19	1.63	1	9	51.1

SD, standard deviation; Min, minimum value; Max, maximum value; CV, coefficient of variation; WT12, weight at 12 mo; CWT, carcass weight; EMA, eye-muscle area; BFT, backfat thickness; MS, marbling score.

**Table 4:** Heritability (diagonal), genetic correlation (above diagonal), and phenotypic correlation (below diagonal) among traits in the study

Trait	WT12	CWT	EMA	BFT	MS
WT12	0.26	0.70	0.33	0.01	−0.14
CWT	0.71	0.35	0.55	0.10	0.17
EMA	0.34	0.54	0.44	−0.24	0.30
BFT	0.17	0.26	0.01	0.46	−0.04
MS	0.01	0.09	0.22	0.06	0.56

WT12, weight at 12 mo; CWT, carcass weight; EMA, eye-muscle area; BFT, backfat thickness; MS, marbling score.

**Table 5:** Comparison of accuracy estimates for traits using whole population in Hanwoo cattle

Trait	r_M1_1)	r_M2_1)				

		ssGBLUP2)		pedBLUP2)		% Accuracy increase
	
		Mean±SD	Range	Mean±SD	Range	
WT123)	0.96	0.45±0.19	0 – 0.95	0.44±0.19	0 – 0.96	2.0
CWT3)	0.95	0.44±0.19	0 – 0.93	0.43±0.18	0 – 0.93	2.5
EMA3)	0.96	0.44±0.19	0 – 0.93	0.42±0.19	0 – 0.93	3.1
BFT3)	0.94	0.43±0.19	0 – 0.93	0.42±0.19	0 – 0.93	3.3
MS3)	0.90	0.41±0.20	0 – 0.93	0.40±0.20	0 – 0.93	4.5

1) r_M1_, Pearson’s correlation between estimated breeding value (EBV) and genomic EBV (GEBV) of individuals; r_M2_, correlation estimates using the inverse of the coefficient matrix from the mixed-model equations (details in Materials and Methods).

2) ssGBLUP, single-step genomic best linear unbiased prediction; pedBLUP, pedigree-based best linear unbiased prediction; SD, standard deviation.

3) WT12, weight at 12 mo; CWT, carcass weight; EMA, eye-muscle area; BFT, backfat thickness; MS, marbling score.

**Table 6:** Comparison of accuracy estimates for traits using info-type in Hanwoo cattle

Info-type1)	Trait2)	r_M1_3)	r_M2_3)				

			ssGBLUP4)		pedBLUP4)		% Accuracy increase
	
			Mean±SD	Range	Mean±SD	Range	
PHEN	WT12	0.99	0.63±0.02	0.45–0.80	0.63±0.02	0.45 – 0.80	0
	CWT	0.98	0.57±0.05	0.41–0.79	0.56±0.05	0.39 – 0.79	1.8
	EMA	0.98	0.52±0.08	0.30–0.80	0.52±0.09	0.26 – 0.80	0
	BFT	0.96	0.51±0.09	0.26–0.80	0.50±0.10	0.20 – 0.81	2.0
	MS	0.96	0.51±0.11	0.23–0.82	0.51±0.11	0.13 – 0.83	0
GEN	WT12	0.89	0.66±0.07	0.30–0.95	0.61±0.09	0 – 0.96	8.1
	CWT	0.91	0.66±0.08	0.32–0.93	0.60±0.11	0 – 0.93	9.0
	EMA	0.92	0.67±0.10	0.34–0.93	0.61±0.15	0 – 0.93	10.2
	BFT	0.9	0.67±0.11	0.34–0.93	0.61±0.16	0 – 0.93	10.7
	MS	0.92	0.70±0.11	0.32–0.93	0.63±0.17	0 – 0.93	10.7
PH+GEN	WT12	0.91	0.68±0.05	0.50–0.95	0.63±0.06	0.38 – 0.95	6.5
	CWT	0.92	0.68±0.06	0.47–0.92	0.63±0.08	0.33 – 0.92	7.2
	EMA	0.93	0.69±0.09	0.43–0.92	0.64±0.13	0.29 – 0.91	8.0
	BFT	0.91	0.69±0.10	0.40–0.92	0.64±0.15	0.25 – 0.91	8.5
	MS	0.93	0.73±0.10	0.43–0.92	0.67±0.16	0.27 – 0.92	8.0

1) PHEN, phenotyped-only bull; GEN, genotyped-only bull; PH+GEN, phenotyped and genotyped bull.

2) WT12, weight at 12 mo; CWT, carcass weight; EMA, eye-muscle area; BFT, backfat thickness; MS, marbling score.

3) r_M1_, Pearson’s correlation between estimated breeding value (EBV) and genomic EBV (GEBV) of individuals; r_M2_, correlation estimates using the inverse of the coefficient matrix from the mixed-model equations (details in Materials and Methods).

4) ssGBLUP, single-step genomic best linear unbiased prediction; pedBLUP, pedigree-based best linear unbiased prediction; SD, standard deviation.

**Table 7:** Comparison of accuracy estimates 1 for traits using bull-type in Hanwoo cattle

Bull-type3)	Trait4)	r_M1_	r_M2_1)				

			ssGBLUP2)		pedBLUP2)		% Accuracy increase
	
			Mean±SD	Range	Mean±SD	Range	
YBULL	WT12	0.71	0.57±0.02	0.48–0.65	0.47±0.03	0.42–0.55	21.3
	CWT	0.70	0.55±0.02	0.46–0.64	0.43±0.03	0.35–0.49	27.9
	EMA	0.73	0.55±0.03	0.45–0.65	0.41±0.04	0.29–0.48	34.1
	BFT	0.75	0.54±0.03	0.44–0.65	0.41±0.04	0.27–0.48	31.7
	MS	0.71	0.57±0.03	0.46–0.66	0.42±0.04	0.28–0.49	35.7
CBULL	WT12	0.96	0.62±0.18	0–0.89	0.60±0.17	0–0.88	3.3
	CWT	0.96	0.60±0.17	0–0.87	0.57±0.16	0–0.86	5.3
	EMA	0.95	0.59±0.17	0–0.87	0.55±0.17	0–0.85	7.3
	BFT	0.91	0.59±0.17	0–0.87	0.54±0.18	0–0.85	9.3
	MS	0.91	0.55±0.21	0–0.88	0.49±0.20	0–0.87	12.2
PBULL	WT12	0.96	0.74±0.18	0–0.95	0.72±0.19	0–0.96	2.8
	CWT	0.96	0.73±0.16	0–0.93	0.71±0.17	0–0.93	2.8
	EMA	0.95	0.73±0.16	0–0.93	0.70±0.17	0–0.93	4.3
	BFT	0.93	0.73±0.16	0–0.93	0.70±0.17	0–0.93	4.3
	MS	0.89	0.70±0.19	0–0.93	0.66±0.22	0–0.93	6.1

1) r_M1_, Pearson’s correlation between estimated breeding value (EBV) and genomic EBV (GEBV) of individuals; r_M2_, correlation estimates using the inverse of the coefficient matrix from the mixed-model equations (details in Materials and Methods).

2) ssGBLUP, single-step genomic best linear unbiased prediction; pedBLUP, pedigree-based best linear unbiased prediction; SD, standard deviation.

3) BULL, young bull-calves; CBULL, young candidate bulls (and calves); PBULL, proven bulls.

4) WT12, weight at 12 mo; CWT, carcass weight; EMA, eye-muscle area; BFT, backfat thickness; MS, marbling score.
